# Validation of the German Normalisation Process Theory Measure G-NoMAD: translation, adaptation, and pilot testing

**DOI:** 10.1186/s43058-023-00505-4

**Published:** 2023-10-16

**Authors:** Johanna Freund, Alexandra Piotrowski, Leah Bührmann, Caroline Oehler, Ingrid Titzler, Anna-Lena Netter, Sebastian Potthoff, David Daniel Ebert, Tracy Finch, Juliane Köberlein-Neu, Anne Etzelmüller

**Affiliations:** 1 Professorship Psychology & Digital Mental Health Care, Department of Sport and Health Sciences, Technical University of Munich, Georg-Brauchle-Ring 60/62, Munich, 80992 Germany; 2 Department of Clinical Psychology and Psychotherapy, Institute of Psychology, Friedrich-Alexander-Universität Erlangen-Nürnberg, Erlangen, Germany; 3 Center for Health Economics and Health Services Research, Schumpeter School of Business and Economics, University of Wuppertal, Wuppertal, Germany; 4 Chair of General Practice II and Patient Centredness in Primary Care, Institute of General Practice and Primary Care (IAMAG), Faculty of Health, Witten/Herdecke University, Witten, Germany; 5 Department of Social Work, Education and Community Wellbeing, Northumbria University, Newcastle Upon Tyne, UK; 6 German Depression Foundation, Leipzig, Germany; 7 Department of Psychiatry and Psychotherapy, University of Leipzig Medical Center, Leipzig, Germany; 8 Department of Clinical Psychology and Psychotherapy, Institute of Psychology, Philipps University of Marburg, Marburg, Germany; 9 Department of Nursing, Midwifery and Health, Northumbria University, Newcastle Upon Tyne, UK; 10 HelloBetter, GET.ON Institut für Online Gesundheitstrainings GmbH, Berlin, Germany

**Keywords:** Implementation Science, Psychometrics, Normalisation process theory, NPT, NoMAD, Instrument development, Implementation outcomes, Validation

## Abstract

**Background:**

Implementing evidence-based healthcare practices (EBPs) is a complex endeavour and often lags behind research-informed decision processes. Understanding and systematically improving implementation using implementation theory can help bridge the gap between research findings and practice. This study aims to translate, pilot, and validate a German version of the English NoMAD questionnaire (G-NoMAD), an instrument derived from the Normalisation Process Theory, to explore the implementation of EBPs.

**Methods:**

Survey data has been collected in four German research projects and subsequently combined into a validation data set. Two versions of the G-NoMAD existed, independently translated from the original English version by two research groups. A measurement invariance analysis was conducted, comparing latent scale structures between groups of respondents to both versions. After determining the baseline model, the questionnaire was tested for different degrees of invariance (configural, metric, scalar, and uniqueness) across samples. A confirmatory factor analysis for three models (a four-factor, a unidimensional, and a hierarchical model) was used to examine the theoretical structure of the G-NoMAD. Finally, psychometric results were discussed in a consensus meeting, and the final instructions, items, and scale format were consented to.

**Results:**

A total of 539 health care professionals completed the questionnaire. The results of the measurement invariance analysis showed configural, partial metric, and partial scalar invariance indicating that the questionnaire versions are comparable. Internal consistency ranged from acceptable to good (0.79 ≤ *α* ≤ 0.85) per subscale. Both the four factor and the hierarchical model achieved a better fit than the unidimensional model, with indices from acceptable (SRMR = 0.08) to good (CFI = 0.97; TLI = 0.96). However, the RMSEA values were only close to acceptable (four-factor model: χ2164 = 1029.84, RMSEA = 0.10; hierarchical model: χ2166 = 1073.43, RMSEA = 0.10).

**Conclusions:**

The G-NoMAD provides a reliable and promising tool to measure the degree of normalisation among individuals involved in implementation activities. Since the fit was similar in the four-factor and the hierarchical model, priority should be given to the practical relevance of the hierarchical model, including a total score and four subscale scores. The findings of this study support the further usage of the G-NoMAD in German implementation settings.

**Trial registration:**

Both the AdAM project (No. NCT03430336, 06/02/2018) and the EU-project ImpleMentAll (No. NCT03652883, 29/08/2018) were registered on ClinicalTrials.gov. The ImplementIT study was registered at the German Clinical Trial Registration (No. DRKS00017078, 18/04/2019). The G-NoMAD validation study was registered at the Open Science Framework (No7u9ab, 17/04/2023).

**Supplementary Information:**

The online version contains supplementary material available at 10.1186/s43058-023-00505-4.

Contributions to the literature

- Pragmatic quantitative measures are powerful tools facilitating the implementation of evidence-based healthcare practices by enabling the assessment and monitoring of implementation outcomes, making the evaluation of implementation processes easier and comparable. Instruments with good psychometric properties are particularly worthwhile, as they could act as early indicators of implementation success, allowing for early identification of challenges and targeted adjustments in the implementation process.
- This study translates, adapts, and validates a German version of the NoMAD questionnaire (G-NoMAD), an instrument to assess normalisation as an implementation outcome.
- The G-NoMAD is a reliable and promising tool to measure the degree of normalisation among health care professionals involved in German implementation settings.
- This study provides suggestions to other researchers who want to translate and validate an (implementation) questionnaire into their language. We also propose an approach for synchronizing different versions in case research teams become aware of each other during their individual research processes.

## Introduction

Implementing evidence-based healthcare practices (EBPs) is a complex endeavour [1] and often lags behind research-informed decision processes [2, 3]. Successful implementation of EBPs is a necessary pre-requisite for optimal and state-of-the-art healthcare provision [4]. Understanding and systematically improving implementation can help close the gap between research findings and practice. Implementation processes and outcomes can be understood and, subsequently, improved using implementation theory [5]. Moreover, such theories can explain change processes in complex systems, including the perspective of multiple stakeholders [6].

Similarly, pragmatic quantitative measures to reliably assess and monitor implementation processes are powerful tools to facilitate the implementation of EBP [7]. Specifically, the valid assessment and evaluation of implementation outcomes, regardless of the observed effect of an EBP, can advance understanding of the underlying mechanisms of implementation by capturing and comparing implementation outcomes and constructs [4]. Using well-developed implementation outcome measures can also be helpful when EBPs do not show the anticipated effect and mediating and moderation effects on the implementation process are explored. Valid and reliable measurement tools can adequately examine implementation strategies and influences on implementation success. Therefore, quantitative measurements are critical to advancing knowledge in implementation research. However, in a systematic review of instruments assessing implementation outcomes by Lewis et al. [8], the authors found that psychometric evidence is lacking and, when available, questionnaires were often of poor psychometric quality. A systematic review of German-language questionnaires assessing implementation constructs and outcomes yielded similar results, indicating an urgent need for valid and reliable German-language measurement tools [9].

## Normalisation Process Theory

The Normalisation Process Theory (NPT) [6, 10, 11] is a vigorously developed, thoroughly tested, and refined medium-range theory, that provides a basis for understanding relevant processes and work that needs to be done to implement an intervention [10]. NPT can be used to understand the dynamics of implementing new practices or interventions in routine health care [10]. The theory postulates that “practices become routinely embedded in social contexts (‘normalised’) as the result of people working, individually and collectively, to enact them” ([11], p. 2). NPT posits four mechanisms—*coherence* (CO), *cognitive participation* (CP), *collective action* (CA), and *reflexive monitoring* (RM)—which promote or inhibit the implementation of complex interventions into routine health care systems [6, 10–12], see details in Table 1. The theory has been widely used for qualitative analyses of implementation activities in various health care contexts [6]. The four mechanisms or core constructs of NPT have been found to be stable across contexts, EBPs, and stakeholders or users [13]. As these constructs can also be used to investigate the potential of practices to become part of daily work [14], i.e. to normalise, NPT is a valuable basis to inform implementation outcome measurement.

**Table 1:** NPT mechanisms following Finch et al. [12]

**Construct**	**Definition: The process which promotes or inhibits…**
*Coherence*	… the sense-making of an innovation to its users. These mechanisms are activated by participants’ investments in meaning.
*Cognitive participation*	… users’ engagement and legitimisation of a practice. These processes are fuelled by participants’ investments of commitment.
*Collective action*	… the enactment of an innovation by its users. These processes are energized by participants’ investments of endeavour.
*Reflexive monitoring*	… users’ understanding of the implications of a practice. These processes are stimulated by participants’ investments in assessment and valuation.

## The NoMAD questionnaire

The “Normalisation Process Theory Measure” NoMAD [12] is an NPT-based questionnaire for assessing and monitoring the implementation process. The development of the questionnaire, which included consensus workshops, cognitive interviews, appraisal of item quality, and expert rating, is described in detail elsewhere [11, 15]. Following the initial development of the NoMAD, Finch et al. [12] conducted initial psychometric tests to establish its reliability and validity. Their results are based on 413 surveys submitted by staff involved in one of six implementation projects across a range of interventions in different settings. A confirmatory factor analysis (CFA) confirmed the theoretical structure of the four NPT constructs, and a test of internal consistency supported the use of the 20 items to measure a general construct of normalisation (*α* = 0.89) as well as a measure of four related constructs (*α* = 0.65–0.81). The NoMAD stands out among other measures in the field, whose psychometric properties are often rated as poor to moderate or for which no information on psychometric properties is available [8].

It is crucial to have language-specific questionnaire versions to capture the perspective of the healthcare workers involved at the local organisation. Having a consistent and validated version in the specific language is important and prevents the coexistence of multiple translations. At the same time, the validation of a translated instrument contributes to improved reliability of the measurements and ensures that the meaning of the original items is retained. The NoMAD questionnaire has been used and validated in different languages and settings. A Dutch translation of the NoMAD questionnaire was validated with a sample of 262 healthcare professionals in the early stages of adopting e-mental health in their occupational tasks [16]. The results showed acceptable internal consistency (0.62 ≥ Cronbach’s alpha ≤ 0.85), and the theorised four-factor structure was mostly confirmed. To facilitate interpretation, they proposed a hierarchical model in which a second-level factor was added to account for the correlation among the four first-level factors. While this approach yielded marginally inferior results concerning the model fit, it could be helpful for the practical application of the NoMAD, as it allows researchers to also use a total score that combines the four NPT constructs.

In addition, the NoMAD was translated into Swedish and validated. After the exclusion of three items, the four-factor model could be successfully replicated, and the four factors yielded good internal consistency (0.78 ≥ Cronbach’s alpha ≤ 0.83) [17]. Further NoMAD translations into Brazilian Portuguese [18] and Chinese [19] demonstrated good internal consistency for all constructs, confirming that translations into other languages are possible while maintaining the psychometric properties. A German version of the NoMAD questionnaire has not yet been psychometrically validated.

## Research aim

Therefore, this study aimed to translate, adapt, and validate a German version of the English NoMAD questionnaire (G-NoMAD), a measurement instrument to assess normalisation as an implementation outcome, in different German health care settings across four projects. Our aims were (1) to assess the internal consistency and the relationships between NPT constructs and (2) to confirm a four factor structure with acceptable model fit according to the theoretical development of the measure along the four NPT concepts.

## Methods

### Study design

**Fig. 1:**
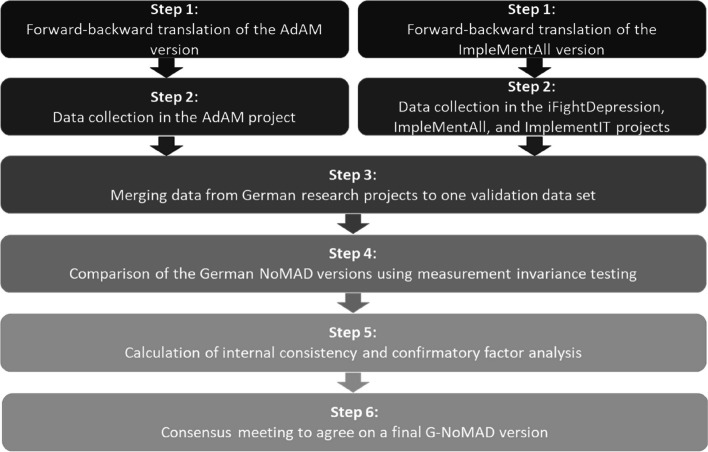
Multi-step procedure for the translation and validation of the G-NoMAD

A multi-step approach, including a forward-backward translation process, an investigation of the theoretical factor structure, and a consensus meeting, was used to translate and validate a German version of the NoMAD questionnaire. All steps are shown in Fig. 1 and explained in more detail in the following.

### Original NoMAD questionnaire

The original NoMAD in the English language consists of three sections: Section A assesses general information about the participant, section B includes three general items on the intervention answered on an 11-point Likert scale ranging from 0 to 10 with descriptive anchors at 0, 5, and 10 ((1)“How familiar does [the intervention] feel for you?”; (2) “Do you feel that [the intervention] is currently a normal part of your work?”; (3) “Do you feel that [the intervention] will become a normal part of your work?”). Section C contains 20 items representing the four key constructs of NPT: *coherence* (4 items), *cognitive participation* (4 items), *collective action* (7 items), and *reflexive monitoring* (5 items). Section C items are answered on a 5-point Likert scale (Option A: 1 = strongly agree; 5 = strongly disagree) or, alternatively, as not relevant with three different answer options (Option B: “not relevant to my role”, “not relevant at this stage”, or “not relevant to the intervention”). Furthermore, the NoMAD shows a clear factor structure and a strong internal consistency supporting a measure to assess normalisation in total (20 items, Cronbach’s *α* = 0.89) as well as for the four subscales (Cronbach’s *α* ranging from 0.65 to 0.81) [12].

### Translation process and pre-testing

Two slightly different versions of the German NoMAD existed, independently translated from the original English version [12] by two research groups [20, 21].

#### AdAM [Anwendung digital-gestütztes Arzneimitteltherapie- und Versorgungs-Management] version

A German translation of the NoMAD was developed within three professional forward and backward translations, a recommended method for translating instruments [22], evaluated separately by three independent researchers using a scoring system. Indifferent points were then discussed within the research team. The research team reviewed the resulting first NoMAD draft. In this step, project-specific adjustments were made to the wording of individual items without changing their meaning. This was followed by a pre-test with physicians, researchers, and members of family physician associations with the opportunity to provide feedback on understanding and wording. The final version was used in a written survey conducted during the AdAM project [20].

#### ImpleMentAll version

Another German translation of the NoMAD was developed in the EU project ImpleMentAll [21] and further used in two German implementation studies [23, 24]. A translation protocol [25] was used in the ImpleMentAll study to ensure a consistent approach across study sites for translating the NoMAD questionnaire into different languages. According to the translation protocol, this was done using a forward-backward translation process by independent translators where discrepancies between the original English version and the back-translated English version were analysed in a structured way and discussed with the original author Tracy Finch. Changes were then integrated into the target language version. All changes have been reported and explained.

#### Version comparisons

Despite different versions, the two translations largely match (see Additional file 1). While the ImpleMentAll version tended to use more technical and scientific terms and was formulated in a more general way, the language style used in the AdAM version was more colloquial and adapted to the specific context. For example, “usual ways of working” was replaced by “previous medication management” to reflect the AdAM study context. The ImpleMentAll version was intended for use in various study sites and the terms were therefore formulated more generally. In both questionnaire versions, items are answered on a 5-point Likert scale (1 = strongly agree; 5 = strongly disagree) and, unlike the original questionnaire, do not include the three different “not relevant” response options (described above) which have been used for the development of the original NoMAD questionnaire [12].

### Data collection

The included data were collected in four implementation projects across five organisations that have used a German version of the NoMAD questionnaire at that time. Of the four projects, one was conducted in the primary care setting (*AdAM*) and three in the context of mental health care (*iFightDepression Marburg, ImpleMentAll, and ImplementIT*). Data was collected through an online-survey (*ImpleMentAll, ImplementIT*) or a survey via paper-pencil (*AdAM, iFightDepression Marburg*). Demographics and background information on the setting were captured to complement the NoMAD data.

#### Organisation 1: AdAM

In AdAM, a clinical decision support system (CDSS) addressing the medication management of patients with polypharmacy was implemented in primary care practices in Germany [20]. The primary analysis was a stepped-wedge cluster randomised controlled trial (C-RCT) to examine the effectiveness of the intervention regarding patient-related outcomes (hospitality and death). The additional survey aimed to gather standardised information on the resources and characteristics of the primary care practices and the way of implementation. General practitioners (GPs) from the C-RCT practices were asked to participate in the survey after all practices had switched to the intervention group. Data were collected from September to December 2020.

#### Organisation 2: iFightDepression Marburg

In the “iFightDepression Marburg” project, the implementation of the internet-based self-management tool “iFightDepression” (iFD; https://tool.ifightdepression.com/) was monitored. The tool is rooted in the principles of Cognitive Behavioural Therapy [26, 27] and can be applied as a supplement to regular depression treatment or to bridge the waiting period. The tool includes six weekly online workshops about specific topics regarding depressive symptoms, including written information, worksheets, exercises, and a mood rating [28]. GPs and psychotherapists who identified patients and provided access to the tool were eligible to participate in the study. The survey was conducted after six one-time information sessions on the iFD tool. Data collection took place from February to November 2018.

#### Organisations 3 and 4: ImpleMentAll project

The German institutions German Depression Foundation (DF) and GET.ON institute (www.geton-institut.de/www.hellobetter.de) were local implementation sites within the EU project “ImpleMentAll” (www.implementall.eu) [21, 29]. This project aimed to examine the effectiveness of tailored implementation (i.e. the ItFits-toolkit) compared to the usual implementation of internet-based interventions (IBIs) based on Cognitive Behavioural Therapy in routine care in twelve sites from nine countries. Data from the two German trial sites at wave 2 (September to November 2018) were used for this analysis.

#### Organisation 3: German Depression Foundation

The nationwide implementation of iFD (see Organisation 2) was aimed for. In press releases, face-to-face and online training and through social media activities, DF tried to inform guides and patients across Germany about iFD. Study participants were iFD guides who provided access to the tool in routine care as well as staff members of DF involved in the technical support and dissemination of iFD.

#### Organisation 4: GET.ON institute

Seven guided IBIs were implemented by the social insurance for agriculture, forestry, and horticulture (SVLFG, www.svlfg.de) to prevent depression among their insured members in selected pilot areas as part of the project “With us in balance” [23]. Staff involved in the counselling on the preventive services (e.g. field workers, in-house staff, and call centre agents) were recruited via kick-off events or supervisors of the respective occupational group.

#### Organisation 5: ImplementIT

As part of the German national depression prevention programme for farmers, gardeners, and foresters, the SVLFG implemented guided, tailored IBIs, and personalised tele-based coaching for their insured members according to a stepwise rollout [23]. The IBIs were provided by the GET.ON institute (www.geton-institut.de/www.hellobetter.de), the personalised tele-based coaching by the company IVPNetworks (www.ivpnetworks.de). Data was collected from April to June 2019.

### Data analysis

All analyses were conducted using the statistical open-source programme R (R 3.6.0 GUI 1.70 El Capitan build, and RStudio Inc., 2018, Version 1.1.463) with packages “psych” (1.8.12) and “lavaan” (0.6–5).

### Descriptive statistics

Response to the questionnaire was analysed, including the total number of responders, corresponding response rates, the total completion of NoMAD items (items 1–20), and the basic completion rate (i.e. all responders that completed one or more items). Respondents’ demographics were calculated, including age, gender, occupation, and work experience.

Mean scale scores were calculated per study site for each NoMAD construct (coherence, cognitive participation, collective action, and reflexive monitoring). Internal consistency was assessed by computing Cronbach’s alpha for each subscale. Cronbach’s alpha was interpreted as acceptable if 0.7 ≥ *α* < 0.8, good if 0.8 ≥ *α* < 0.9, and excellent if *α* ≥ 0.9 [30]. Correlations were calculated between the NoMAD constructs for the pooled sample.

### Confirmatory factor analysis

A CFA was performed to verify the factor structure of the NoMAD questionnaire. As theory suggests, the NoMAD has a four-factor structure (coherence, cognitive participation, collective action, and reflexive monitoring). Accordingly, the four-factor model was used in the CFA. Additionally, a unidimensional as well as a hierarchical model were computed. The hierarchical model represents the idea of a global NoMAD score (i.e. a total normalisation score) consisting of four sub-scores. For all models, the data were fitted on the predefined model structure. For evaluating model fit, the fit indices Comparative Fit Index (CFI), Tucker Lewis Index (TLI), Root Mean Square Error of Approximation (RMSEA), and Standardised Root Mean Square Residual (SRMR) were interpreted. Conservative cut-off scores for acceptable fit were applied as suggested by the literature [31–34]. A cut-off value of 0.4 was chosen to evaluate the factor loadings, where values below 0.4 indicated a low item loading on the latent construct [35], and items loading below 0.2 were considered insufficient.

### Measurement invariance testing

We investigated whether the NoMAD instrument is measurement invariant across two samples representing data from respondents to two different versions of the German translation of the NoMAD. The analysis followed the 4-step approach of conducting measurement invariance testing with ordinal survey data as described by Bowen and Masa [36]. First, a CFA was performed to estimate a baseline model in both groups (see above). Given the ordinal nature of the data, the robust option of the diagonally weighted least square (WLSMV) estimator was used to examine the expected dimensionality of the instrument scale [37]. The chi-square test statistic (*χ*^2^) was reported. However, due to its sensitivity to sample size and violation of the normality assumption [38], descriptive model fit indices were used to evaluate the model fit. The CFI, TLI, RMSEA, and SRMR are reported and interpreted. After determining the baseline model, the questionnaire was tested for different degrees of invariance (configural, metric, scalar, and uniqueness) across samples. Parameters of the models (i.e. factor loadings, thresholds, and residuals) were progressively constrained across groups to investigate to what degree the instrument can be interpreted as invariant between groups [39]. At the *configural* invariance level, the form of the factor model was compared across groups [40]. No parameters are restricted between groups beyond fixing the first loading of each factor to 1 as a referent indicator. If the unconstrained multiple group model meets fit criteria [41], the analysis continues to test the factor structure for *metric* invariance. Scales with *metric* invariance have statistically equivalent factor loadings across groups [40]. All factor loadings are constrained to be equal, and the resulting model fit is compared to the fit of the *configural* model. If the difference between the model fit is not significant (ΔCFI ≤ 0.1) [42], the testing will proceed to explore *scalar* invariance. If the difference between the models is significant (ΔCFI ≤ 0.1), most variant parameters will be set free. If the number of freed parameters is below 20% of the total number of parameters, the testing is continued [40]. In the next step, factor loadings and thresholds are constrained across groups. The same criteria—i.e. ΔCFI ≤ 0.1 [42] and the 20% rule [40]—as applied in the previous steps are evaluated. *Scalar* invariance is generally considered the minimum level of invariance to be able to interpret scores equally across groups [36]. *Uniqueness* invariance is investigated by constraining all residual variances across the groups. However, this level of invariance is usually not reached—and not deemed necessary—within measurement invariance testing [40].

### Consensus meeting

A 4-h consensus meeting was held (1) to review the psychometric results of the questionnaire, (2) to review the two different versions of the German translation of the NoMAD, (3) to consent to the final scale format, (4) to discuss instructions, and (5) to decide whether the option “not applicable” should be used for the German NoMAD version as well, which would be in line with the original questionnaire. Researchers responsible for the survey instruments of each of the four projects were invited via email to participate. Due to the COVID-19 pandemic, the consensus meeting was held online. Ten researchers (AE, AP, CO, CS, IT, JF, JG, JK, LB, and SP) participated. Following the Nominal Group Technique (NGT), a structured group discussion led by one or more moderators (here: AE and AP), participant reflections on the abovementioned five topics were captured, and discussions were provided. More particularly, after a brief introduction to each topic, participants were given time to list their responses to a topic. Next, participants were asked to share their thoughts. Statements were documented in a condensed form and discussed. Finally, participants were asked to vote on their preferred option for a topic discussed. After the consensus meeting, a consented version was applied and documented for final approval by an independent lector. All sub-steps of item adaptation, including discussions and rationale for decisions, were documented (see Additional file 1).

## Results

### Response

Data from four projects across five organisations were used for the analysis (see Table 2). The mean response rate is 55.4% (539 respondents out of 973 invited participants). A total of 539 surveys were used for the analysis.

**Table 2:** Response rates and item completion per organisation

Organisation	Invited	Responded (≥ 1 NoMAD item)	Response rate	Total completion NoMAD items 1–20	Completion rate^a^
Organisation 1: AdAM	750	328	43.7%	292	96.7%
Organisation 2: iFD Marburg	78	77	98.7%	69	99.1%
Organisation 3: DF	21	16	76.2%	16	100%
Organisation 4: GET.ON	49	46	93.9%	46	100%
Organisation 5: ImplementIT	75	72	96.0%	72	100%

*iFD* iFightDepression, *DF* Depression Foundation

^a^Completion rate was assessed among responders (≥ 1 NoMAD item)

### Sample

Table 3 provides an overview of participant characteristics per individual organisation. Most participants were between 51 and 60 years old (*n* = 237, 44.0%), male (*n* = 330, 61.2%), and worked as practice owners (*n* = 309, 57.3%) for more than 10 years in their current organisation (*n* = 333, 61.8%).

**Table 3:** Description of study participants

**Variable**	Organisation 1: AdAM (*n* = 328)	Organisation 2: Marburg (*n* = 77)	Organisation 3: DF (*n* = 16)	Organisation 4: GET.ON (*n* = 46)	Organisation 5: ImplementIT (*n* = 72)	Full sample (*n* = 539)
Age, *n* (%)						
Under 30 years	0 (0.0)	17 (21.8)	4 (25.0)	1 (2.2)	2 (2.8)	24 (4.5)
30–40 years	11 (3.4)	20 (25.6)	4 (19.1)	10 (21.7)	10 (13.9)	55 (10.2)
41–50 years	67 (20.2)	13 (16.9)	4 (25.0)	12 (26.1)	24 (33.3)	120 (22.3)
51–60 years	165 (50.3)	17 (21.8)	3 (18.8)	21 (45.6)	31 (43.1)	237 (44.0)
Over 60 years	82 (25.0)	10 (12.8)	1 (6.3)	2 (4.35)	3 (4.2)	98 (18.2)
NA	3 (1.2)	0 (0.0)	0 (0.0)	0 (0.0)	2 (2.8)	5 (1.0)
Gender						
Male	203 (61.9)	37 (47.4)	4 (25.0)	33 (71.7)	53 (73.6)	330 (61.2)
Female, *n* (%)	125 (38.1)	38 (49.4)	12 (75.0)	13 (28.3)	19 (26.4)	207 (38.4)
Diverse	0 (0)	0 (0.0)	0 (0.0)	0 (0.0)	0 (0.0)	0 (0.0)
NA	0 (0)	2 (2.6)	0 (0.0)	0 (0.0)	0 (0.0)	2 (0.4)
Occupation						
Practice owner	309 (93.1)	0 (0.0)	0 (0.0)	0 (0.0)	0 (0.0)	309 (57.3)
Referrer	0 (0.0)	1 (1.3)	1 (6.3)	29 (63.0)	71 (98.6)	102 (18.9)
General practitioner	0 (0.0)	7 (9.1)	0 (0.0)	0 (0.0)	0 (0.0)	7 (1.3)
Administrative employee	0 (0.0)	0 (0.0)	4 (25.0)	14 (28.6)	0 (0.0)	18 (3.3)
Psychologist, e-coach, psychotherapist	0 (0.0)	62 (80.5)	11 (68.8)	3 (6.1)	0 (0.0)	76 (14.1)
Employed doctor in training	14 (4.2)	0 (0.0)	0 (0.0)	0 (0.0)	0 (0.0)	14 (2.6)
Other health care worker	0 (0.0)	1 (1.3)	0 (0.0)	0 (0.0)	1 (1.4)	2 (0.4)
Nurse	0 (0.0)	2 (2.6)	0 (0.0)	0 (0.0)	0 (0.0)	2 (0.4)
Employed (specialist) doctor	3 (0.9)	3 (3.9)	0 (0.0)	0 (0.0)	0 (0.0)	6 (1.1)
Other practice staff	2 (0.6)	0 (0.0)	0 (0.0)	0 (0.0)	0 (0.0)	2 (0.4)
Support/information and communication technology (ICT) worker	0 (0.0)	1 (1.3)	0 (0.0)	0 (0.0)	0 (0.0)	1 (0.2)
Other employee	0 (0.0)	0 (0.0)	0 (0.0)	0 (0.0)	0 (0.0)	0 (0.0)
NA	0 (0.0)	0 (0.0)	0 (0.0)	0 (0.0)	0 (0.0)	0 (0.0)
Work duration in the organisation						
Less than 1 year	0 (0.0)	17 (22.1)	2 (12.5)	4 (8.7)	0 (0.0)	23 (4.3)
1–2 years	4 (1.2)	12 (15.6)	3 (18.8)	7 (15.2)	1 (1.4)	27 (5.0)
3–5 years	29 (8.8)	11 (14.3)	4 (25.0)	8 (17.4)	1 (1.4)	53 (9.8)
6–10 years	66 (20.1)	11 (14.3)	2 (12.5)	15 (32.6)	8 (11.1)	102 (18.9)
More than 10 years	229 (69.8)	25 (32.5)	5 (31.3)	12 (26.1)	62 (86.1)	333 (61.8)
NA	0 (0.0)	1 (1.3)	0 (0.0)	0 (0.0)	0 (0.0)	1 (0.2)

### Measurement invariance

Two CFA were conducted for group 1, “AdAM version”, and group 2, “ImpleMentAll version”, separately. Slightly better fit indices for the four-factor model are shown in group 1 (*χ*^2^ = 525.754; df = 164, CFI = 0.978; TLI = 0.974; RMSEA = 0.082; SRMR = 0.068) compared to group 2 (*χ*^2^ = 453.500; df = 164, CFI = 0.970; TLI = 0.965; RMSEA = 0.092; SRMR = 0.965). A measurement invariance analysis was performed to show whether the different questionnaire versions capture the same constructs and are, therefore, comparable.

Fit statistics of all invariance levels are illustrated in Table 4. First, we tested for configural invariance (Model 1, M1). The fit indices met our pre-specified criteria, indicating that the two groups share the same configural model. Second, we tested for metric invariance based on a model with constrained factor loadings across the two groups (M2). A comparison of M1 and M2 showed a change of the CFI fit of more than 0.01, and thus, M2 was rejected. However, after freeing the factor loadings for the second and third items within the factor collective action (CA.2, CA.3), a partial metric invariance model (M2a) was tested since these thresholds differed between the groups. Due to a change in the CFI score below 0.01, M2a was accepted. Third, scalar invariance was investigated using a model with constrained factor loadings and thresholds across the two groups (M3). A comparison of M2a and M3 showed again a change of the CFI fit of more than 0.01, and thus, M3 was rejected. After freeing the factor loadings for items “CA.2” and “CA.3” as well as the thresholds “RM.4|t2” and “CO.4|t3”, a partial scalar invariance model (M3a) was tested, indicating an acceptable model fit. Since the results of measurement invariance indicate that the questionnaire versions are comparable, the results are reported jointly for both questionnaire versions in the following.

**Table 4:** Results of the measurement invariance analysis

**Model**	*χ*^2^	df	CFI	TLI	RMSEA	SRMR	∆ *χ*^2^	∆ CFI	Model comparison	Decision
**M1:** Configural invariance	977.413	328	0.975	0.971	0.086	0.075	–	–	–	Accept
**M2:** Metric invariance	1363.908	344	0.960	0.956	0.105	0.105	386.495	0.015	M1	Reject
**M2a:** Partial metric invariance^a^	1163.615	342	0.968	0.964	0.095	0.095	186.202	0.007	M1	Accept
**M3:** Scalar invariance	1491.756	398	0.957	0.959	0.101	0.077	328.141	0.011	M2a	Reject
**M3a:** Partial scalar invariance^b^	1429.600	396	0.960	0.961	0.099	0.077	265.985	0.008	M2a	Accept

*CFI* Comparative Fit Index, *TLI* Tucker Lewis Index, *RMSEA* Root Mean Square Error of Approximation, *SRMR* Standardised Root Mean Square Residual

^a^Freed: factor loadings for CA.2 and CA.3

^b^Freed: factor loadings for CA.2 and CA.3 and thresholds RM.4|t2 and CO.4|t3

### Scale scores

The mean scale scores per organisation are presented in Table 5. In the pooled sample, item responses in the NPT constructs *coherence* and *cognitive participation* tend to agree, while *collective action* and *reflexive monitoring* instead received neutral answers. In addition, the responses to items vary the least for *collective action* and the most for *cognitive participation*.

**Table 5:** NoMAD-G mean scale scores per organisation

Scale	Organisation					Full sample
	1	2	3	4	5	
*N*	328	328	16	46	72	539
CO	3.35 (0.98)	2.67 (0.53)	3.70 (0.70)	4.00 (0.72)	3.75 (0.68)	3.52 (0.92)
CP	3.44 (1.01)	2.57 (0.71)	3.98 (0.99)	4.25 (0.67)	4.00 (0.60)	3.48 (1.01)
CA	3.16 (0.73)	2.59 (0.49)	3.65 (0.69)	3.78 (0.70)	3.72 (0.53)	3.23 (0.75)
RM	3.07 (0.88)	2.60 (0.40)	3.86 (0.70)	3.56 (0.71)	3.55 (0.61)	3.14 (0.84)

*CO* Coherence, *CP* Cognitive participation, *CA* Collective action, *RM* Reflexive monitoring

### Internal consistency

Cronbach’s alpha was computed for each subscale. The internal consistency ranges from “acceptable” for *collective action* and *reflexive monitoring* (each *α* = 0.79) to “good” for *coherence* and *cognitive participation* (each *α* = 0.85). Overall, the NoMAD scale comprising all 20 items is highly reliable (*α* = 0.93).

### Relationships between NPT constructs

All correlations between the four NPT construct measures are shown in Table 6. The highest correlation between the NoMAD constructs could be identified for *coherence* and *cognitive participation* (*r* = 0.76) and the lowest for *coherence* and *collective action* (*r* = 0.64). This indicates a high level of correlation for summated NoMAD scores [43].

**Table 6:** Correlations between NoMAD constructs (factors)

Scale	Coherence	Cognitive participation	Collective action	Reflexive monitoring
Coherence	1			
Cognitive participation	.76	1		
Collective action	.64	.72	1	
Reflexive monitoring	.69	.71	.76	1

### Factor structure

The CFA results and related fit indices are presented in Table 7, including the first order four-factor model that defines normalisation as four correlated constructs, the first-order unidimensional model, and the hierarchical model. In the latter, it is assumed that a second-level factor explains the correlations between the four first-level factors. Both the four factor model and the hierarchical model achieved a better fit than the unidimensional model with indices from acceptable (SRMR = 0.08) to good (CFI = 0.97; TLI = 0.96). However, the RMSEA value of both models is only close to acceptable (four-factor model: χ2164 = 1029.84, RMSEA = 0.10; hierarchical model: χ2166 = 1073.43, RMSEA = 0.10). Since the fit is similar in both models, priority should be given to the practical relevance of the hierarchical model, which includes a total score and subscale scores.

**Table 7:** Confirmatory factor analysis (CFA). Modified models are included in the CFA to explore potential improvements

Model	*χ*^2^	df	CFI	TLI	RMSEA	SRMR
Four-factor	1029.84	164	0.97	0.96	0.10	0.08
Unidimensional	1357.95	170	0.96	0.95	0.11	0.09
Hierarchical	1073.43	166	0.97	0.96	0.10	0.08
Hierarchical modified 1^a^	972.17	148	0.97	0.97	0.10	0.08

*CFI* Comparative Fit Index, *TLI* Tucker Lewis Index, *RMSEA* Root Mean Square Error of Approximation, *SRMR* Standardised Root Mean Square Residual

^a^Hierarchical model without item “RM1”

### Potential model improvements

Potential model improvements were investigated for the hierarchical model. Based on the factor loadings in the CFA, it can be assumed that item RM.1 (“*I am aware of reports about the effects of [the intervention].*”) has a weak relationship with the superordinate construct RM (*λ* = 0.12). Thus, item RM.1 was removed, and the modified four-factor model showed a slightly better fit than the previous model (see Table 7).

### Consensus version of the G-NoMAD

A consensus version was produced, presenting the final German version of the NoMAD, termed G-NoMAD (see Additional file 2). The wording of the response scale was consented to, and the accompanying instructions were adapted. Finally, the consensus group agreed on the renewed inclusion of the option “not applicable”, which, contrary to the original NoMAD version, was not previously applied for in either German questionnaire versions. This decision was motivated by methodological discussions on the advantages and disadvantages of this answer option [44–46] and the results showing that a tendency toward the middle (if an item was not applicable, the middle/neutral position “3” should still be chosen) was evident within the analysed data, which may bias interpretation.

## Discussion

The “Normalisation Process Theory Measure” questionnaire (NoMAD) is a theoretically derived instrument for measuring factors relevant to the implementation of interventions that transform the existing work practices of individuals [12, 15]. Since its development, the NoMAD has been translated from the original English and used in multiple languages across different countries, settings, and studies [16–19]. The current study aimed to review several German translations and pilot applications, validate the instrument, and publish an official German-language version of the NoMAD questionnaire for research and practice purposes.

### Main findings

The G-NoMAD instrument showed good psychometric properties to capture perceptions of individuals involved in implementation activities in different German-speaking intervention studies and settings. Tests of internal consistency confirmed the validity of an overall measure of “normalisation” (20 items, *α* = 0.93), as well as the four separate NPT constructs coherence, cognitive participation, collective action, and reflexive monitoring (*α* = 0.79–0.85). Correlations between the four NPT construct measures can be considered as high (ranging from *r* = 0.64–0.76). Using CFA, the hypothesised four-factor structure was largely confirmed, as all fit indices (except for the RMSEA value) were found to be acceptable to good. Since the fit to the observed data was similar in the four-factor and the hierarchical model, priority should be given to the practical relevance of the hierarchical model for users in research and practice, which includes a total score and four subscale scores.

### Comparison with previous literature

In line with our findings, results from the original English NoMAD validation study [12] showed a clear factor structure and a strong internal consistency. The internal consistency and the correlations between construct measures were even slightly higher in the present study (Cronbach’s *α* = 0.79–0.85; construct correlations *r* = 0.64–0.76) compared to the validation results of the original measure (Cronbach’s *α* = 0.65–0.81; construct correlations *r* = 0.49–0.68) [12].

The current version of the NoMAD also compares favourably concerning internal consistency and construct correlation against other translations of the measure into Dutch [16], Swedish [17], Brazilian Portuguese [18], and Chinese [19]. In the Dutch NoMAD validation study [16], the four-factor model showed the best fit with the observed data. However, in this study, both the four-factor model and the hierarchical model achieved a similar fit.

While most fit indices in this study can be classified as acceptable (SRMR = 0.08) to good (CFI = 0.97; TLI = 0.96), the RMSEA value of both models was only close to acceptable (RMSEA = 0.10). In contrast to our study, the results of the English [12] and Chinese validation studies showed acceptable psychometric properties across all fit indices (English version: CFI = 0.95, RMSEA = 0.08, SRMR = 0.03, TLI = 0.93; Chinese version: CFI = 0.92, RMSEA = 0.01, SRMR = 0.05, TLI = 0.91). In the Dutch validation study [16], all fit indices were outside the desired thresholds (CFI = 0.90, RMSEA = 0.12, SRMR = 0.11, TLI = 0.88), whereas, in our study, this only applied to the RMSEA value. It should be noted that there are only recommendations for model evaluation and no established guidelines for what constitutes an appropriate fit [38]. Moreover, it is possible for a model to fit the data even though one or more measures of fit indicate a poor fit [38]. In view of this, it can be considered a strength of the present study that, despite the different interventions and settings, largely good psychometric values could be achieved.

### Limitations

First, two slightly different versions of the German NoMAD have been used to validate the questionnaire. While the ImpleMentAll version was formulated in a more general way to consider superordinate contexts of 12 different sites, the language style of the AdAM version is more colloquial and adapted to the specific context. Although the measurement invariance analysis confirmed that the two versions are comparable, this fact limits the validity of the results. At the same time, the results of this study provide a common basis for a unified German NoMAD questionnaire for implementation research and practice in which the study results as well as the experiences from both research groups were taken into account.

Second, unlike the original English NoMAD [12, 15], participants in all involved projects were instructed that if an item was not applicable, the middle/neutral position “3” should still be chosen. This could have led to the confounding of answers with different meanings (e.g. the question was not understood, skipped, interpreted as not applicable, the response was refused or remained unanswered due to ignorance), and the bias of the overall results may be large [45]. In the case of compulsory items, the checkbox might have been only ticked to move on to the next item and to be able to continue with the questionnaire, which could lead to an inflationary use of the “3”. This tendency toward the middle is evident in the ImpleMentAll study across 12 trial sites (mean scores in the range from 3.1 to 4.3, with the majority scattering between 3.5 to 3.7) [29] as well as in this G-NoMAD validation data (mean scores in the range from 2.6 to 4.0 per organisation, with the majority scattering between 3.1 to 3.5). Thus, in our suggested G-NoMAD version (see Additional files 2 and 3), we recommend, in line with the original version of the NoMAD [12, 15], the use of the not applicable option for the items of the questionnaire and to statistically take this into account as a “missing item”. We consider this fall-back category useful to address those possible responders who may not have the ability or characteristic of answering a question or to whom specific questions do not apply (e.g. persons as sole practitioners who cannot provide information on organisational or team-related aspects; persons who are not involved in the entire implementation process, but only in peripheral areas). This fall-back category also provides a usable data point, which gives information about the non-processing of the task or non-answering of a question.

Third, as a further limitation, it must be deduced that using the NoMAD in a study setting may produce different results than in a routine setting without an accompanying evaluation. Fourth, NPT was developed using qualitative research of social processes and actions at an individual and collective level. NoMAD provides a tool to statistically explore the importance of NPT constructs relevant to achieving and maintaining practice change. However, to fully understand people’s perceptions of the complexities of implementation work, it is likely to require a combination of quantitative and qualitative research methods.

### Strengths

A large sample size (*N* = 539) across five study sites was reached in this study, providing a sufficient data basis for the psychometric evaluation of the G-NoMAD. Across all organisations, high response and completion rates have been reported indicating a high acceptance and usability of the questionnaire among participants.

The authors of the original NoMAD described the tool as a “pragmatic measure” of implementation, encouraging users to tailor it to the demands of their respective implementation projects [12]. The current study confirms the flexibility of the measure with regard to its application across a variety of implementation settings and projects (e.g. small practices with one general practitioner and larger organisations with different employed staff roles), including a variety of interventions (e.g. mental health interventions and medication management tools), and involved individuals (e.g. psychologists, general practitioners, and health care workers).

### Future research

Quantitative instruments and validated translations are urgently needed in the field of implementation science. This study provides suggestions to other researchers who want to translate and validate an (implementation) questionnaire into their language or merge different existing versions. Even if the results of this study support the broad usage of the G-NoMAD, the modified translation of the G-NoMAD should be further evaluated concerning its psychometric properties. Additionally, the psychometric sensitivity of NoMAD to longitudinal change [29] and the verification of NoMAD with other instruments measuring implementation outcomes (longitudinally) are yet to be explored. Additionally, the think-aloud method can be used to investigate user experience and thoughts when answering the questionnaire to understand deeper processes.

Results of the AdAM project indicate that the NoMAD questionnaire seems equally feasible/applicable for large organisations and individual settings (e.g. small physician practices with only one practice owner) in which implementation of EBPs is primarily done by one person and collective implementation activities are less obviously occurring. However, this issue should be further explored in future research.

### Practical implications and use of the G-NoMAD

In order to be able to use the G-NoMAD for different implementation contexts, we provide detailed instructions on how to modify the questionnaire for different implementation contexts to improve its usability (see Additional file 3). Additionally, we provide recommendations to adapt the German instruction text to the respective study (e.g. by including descriptions of the projects, the implementation object, and the roles of the individuals involved). We invite to adapt the instrument according to the instruction manual to increase the ease of use in routine settings and to enable higher external validity. Furthermore, recommendations for analysing the “not relevant” option are described in the manual.

## Conclusions

G-NoMAD provides a reliable and promising tool to measure the degree of normalisation among health care professionals and other individuals involved in implementation activities. The findings of this study support the further usage of the G-NoMAD in German-language implementation settings. The measure can be used to statistically explore NPT mechanisms involved in achieving and maintaining practice change. It can also be used alongside qualitative studies. The practical relevance of the hierarchical model has to be emphasised, which includes a total “normalisation” score and four subscale scores.

In our various research projects, we have recognised the importance of such a measurement tool. Through the professional exchange over several projects and the possibility of a validation project, we are glad that we can now provide researchers and practitioners with a basis for further implementation and evaluation.

The research and validation team with expertise in implementation science and practice is happy to be available to answer any questions at the following email address: german.nomad2022@gmail.com.

### Supplementary Information

**Additional file 1.** Questionnaire development.

**Additional file 2.** G-NoMAD questionnaire.

**Additional file 3.** G-NoMAD manual.

## Data Availability

The data that support the findings of this study are available from Universität Erlangen-Nürnberg but restrictions apply to the availability of these data, which were used under licence for the current study, and so are not publicly available. Data are however available from the authors upon reasonable request and with permission of all involved projects/institutions.

## Abbreviations

AdAM: Anwendung digital-gestütztes Arzneimitteltherapie- und Versorgungs-Management
CA: Collective action
CO: Coherence
CFA: Confirmatory Factor Analysis
CFI: Comparative Fit Index
CP: Cognitive participation
C-RCT: Cluster randomised controlled trial
DF: Depression Foundation
EBP: Evidence-based healthcare practice
G-NoMAD: German version of the NoMAD questionnaire
GP: General practitioner
ICT: Information and communication technology
iFD: IFight Depression
NGT: Nominal Group Technique
NoMAD: Normalisation Process Theory Measure
NPT: Normalisation Process Theory
RM: Reflexive monitoring
RMSEA: Root Mean Square Error of Approximation
SRMR: Standardised Root Mean Square Residual
SVLFG: Social insurance for agriculture, forestry and horticulture
TLI: Tucker Lewis Index
WLSMV: Diagonally weighted least squares
