# Supplementary material for: Validation of the German Normalisation Process Theory Measure G-NoMAD: translation, adaptation, and pilot testing
Source: Implement Sci Commun. 2023 Oct 16;4:126. doi: 10.1186/s43058-023-00505-4 (PMC10578017; doi:10.1186/s43058-023-00505-4)
Supplement: Supplementary file 2 — Additional file 2. G-NoMAD questionnaire. [file 43058_2023_505_MOESM2_ESM.docx]

| 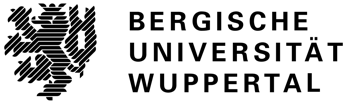 | **Bitte wie folgt zitieren:** Freund, J., Piotrowski, A., Bührmann, L., Oehler, C., Titzler., I., Netter, A., Potthoff, S., Ebert, D. D., Finch, T., Köberlein-Neu, J., Etzelmüller, A. (2023). Validation of the German Normalization Process Theory Measure G-NoMAD: Translation, Adaptation, and Pilot Testing. | 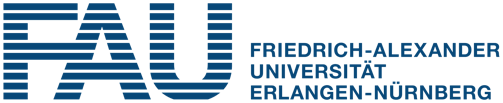 |
| --- | --- | --- |
| 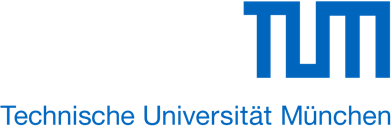 |  | 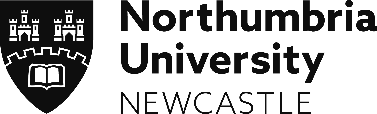 |

**G-NoMAD Fragebogen**

**[BITTE PASSEN SIE DIESEN TEXT AN IHR EIGENES PROJEKT AN. ERSETZEN SIE [die Intervention] MIT IHRER EIGENEN INTERVENTIONS- ODER TÄTIGKEITSBESCHREIBUNG UND LÖSCHEN SIE AM ENDE DIE GELBEN INSTRUKTIONSFELDER VOR DEM FRAGEBOGENEINSATZ.]**

**Diese Befragung soll dazu beitragen, ein besseres Verständnis für die Anwendung und Integration neuer Technologien und komplexer Interventionen im Gesundheitswesen zu erlangen.**In dieser Befragung werden Ihnen Fragen zur Einführung und Anwendung von [der Intervention] gestellt.

[Beteiligte Personen können je nach Kontext unterschiedliche Rollen und Tätigkeitsfelder bei der Einführung und Anwendung [der Intervention] haben. Falls Personen verschiedener Tätigkeitsbereiche an der Befragung teilnehmen oder [die Intervention] auf verschiedene Weise nutzen, empfehlen wir an dieser Stelle wie im Manual beschrieben eine Rollenbeschreibung hinzuzufügen.]

Bitte wählen Sie aus den nachstehenden Aussagen eine Option aus, die **Ihre Hauptrolle** in Bezug auf [die Intervention] am besten beschreibt:

O       Ich bin involviert in das Management / die Verwaltung [der Intervention]

O       Ich bin in die Anwendung/Bereitstellung [der Intervention] involviert

O       Ich bin in die Unterstützung/den Support  [der Intervention] involviert

O       Sonstige Rolle: [Rolle beschreiben; ggf. Freitextfeld zur Spezifikation bereitstellen]

Bitte beantworten Sie alle nachfolgenden Fragen aus der Perspektive dieser Rolle, welche Sie hauptsächlich in Bezug auf [die Intervention] ausüben. Je nach Rolle oder Tätigkeit in Bezug auf [die Intervention] können einige Aussagen für Sie persönlich relevanter sein als andere. Wir bitten Sie trotzdem alle Fragen bestmöglich zu beantworten.

Die Umfrage besteht aus drei Teilen. In Teil A werden einige kurze Fragen zu Ihrer Person und Ihrer Rolle gestellt. Teil B enthält drei allgemeine Fragen zu [der Intervention] in Ihrem speziellen Kontext. Teil C enthält eine Reihe von spezifischen Fragen zu [der Intervention].

Bitte nehmen Sie sich Zeit, um zu entscheiden, welche Antwort am besten zu Ihren Erfahrungen passt, und kreuzen Sie die entsprechende Antwortoption an. Es gibt grundsätzlich keine richtigen oder falschen Antworten.

| **Teil A: *Soziodemographische Angaben und optionale weitere Angaben zur Person/Rolle/Einstellung zur Intervention*** |  |
| --- | --- |
| 1. **Wie alt sind sie? _________ Jahre** 2. **Welchem Geschlecht fühlen sie sich zugehörig?        O weiblich   O männlich  O divers** 3. **Wie lange arbeiten Sie bereits für Ihre Institution? (optional)**   O       Weniger als ein Jahr  O       1-2 Jahre  O       3-5 Jahre  O       6-10 Jahre  O       11-15 Jahre  O       Mehr als 15 Jahre   1. **Welche der folgenden Tätigkeiten in der Versorgung von PatientInnen üben Sie aus, im Rahmen derer Sie [die Intervention] anwenden würden? (optional)**   O       Ich bin PsychologIn, PsychotherapeutIn (in Ausbildung), PsychiaterIn  O       Ich bin ZuweiserIn (z.B. Hausarzt/Hausärztin HausarztIn, SozialarbeiterIn, ApothekerIn, GemeindearbeiterIn oder Case  ManagerIn) und überweise PatientInnen an andere Fachstellen  O       Ich bin KrankpflegerIn  O       Ich bin AdministratorIn, wie Bürokauffrau/-mann  O       Ich bin Informations und Kommunikationstechnologie (IKT)-MitarbeiterIn und z. B. als Sicherheitsbeauftragte(r),  InstandhalterIn oder Helpdesk-MitarbeiterIn tätig.  O       Sonstige: ___________________   1. **Haben Sie schon praktische Erfahrungen mit [der Intervention]? (optional)**   O       Ja  O       Nein     1. **Für wie wahrscheinlich halten Sie es, dass Sie [die Intervention] einem Kollegen/einer Kollegin weiterempfehlen würden? (optional)**   O       Sehr wahrscheinlich  O       Wahrscheinlich  O       Eher wahrscheinlich  O       Eher unwahrscheinlich  O       Unwahrscheinlich  O       Völlig unwahrscheinlich | |

| **Teil B: *Globale NoMAD Fragen*** |
| --- |
|  |

| Wie vertraut fühlt sich [die Intervention] für Sie an? | | | | | | | | | | | | |
| --- | --- | --- | --- | --- | --- | --- | --- | --- | --- | --- | --- | --- |
| Fühlt sich noch sehr neu an | | | |  | | | | | **Fühlt sich vollkommen vertraut an** | | | |
|  | | | |  | | | | |  | | | |
| 0 | 1 | 2 | 3 | | 4 | 5 | 6 | 7 | | 8 | 9 | 10 |
| Betrachten Sie [die Intervention] momentan als einen gewohnten Bestandteil Ihrer Arbeit? | | | | | | | | | | | | |
| Überhaupt nicht | | | | **Etwas** | | | | | **Vollständig** | | | |
|  | | | |  | | | | |  | | | |
| 0 | 1 | 2 | 3 | | 4 | 5 | 6 | 7 | | 8 | 9 | 10 |
| Können Sie sich vorstellen, dass [die Intervention] ein gewohnter Bestandteil Ihrer Arbeit werden wird? | | | | | | | | | | | | |
| Überhaupt nicht | | | | **Etwas** | | | | | **Vollständig** | | | |
|  | | | |  | | | | |  | | | |
| 0 | 1 | 2 | 3 | | 4 | 5 | 6 | 7 | | 8 | 9 | 10 |

| **Teil C: *NoMAD Fragebogen*** |
| --- |
|  |

**Bitte geben Sie im Folgenden an, inwieweit Sie der jeweiligen Aussage auf einer Skala von 1 („Stimme überhaupt nicht zu“) über 3 („weder noch“) bis hin zu 5 („Stimme voll und ganz zu“) zustimmen. Einige Fragen können Sie vielleicht zum jetzigen Zeitpunkt noch nicht beantworten oder sind noch unschlüssig. Geben Sie in diesem Fall bitte “nicht zutreffend” an. Bitte nutzen Sie diese Variante sparsam und geben Sie möglichst oft eine Antwort auf der Skala 1 bis 5 an.**

|  | | **1**  **Stimme überhaupt**  **nicht zu** | **2**  **Stimme nicht zu** | **3**  **Weder noch** | **4**  **Stimme zu** | **5**  **Stimme voll und ganz zu** | **Nicht zutreffend** | |  |
| --- | --- | --- | --- | --- | --- | --- | --- | --- | --- |
| **1.** | **Ich kann nachvollziehen, inwiefern sich [die Intervention] von üblichen Arbeitsweisen unterscheidet.** |  |  |  |  |  | |  | |
| **2.** | **Die Mitarbeitenden dieser Organisation haben ein gemeinsames Verständnis vom Zweck [der Intervention].** |  |  |  |  |  | |  | |
| **3.** | **Ich verstehe, wie [die Intervention] meine Arbeitsweise beeinflusst.** |  |  |  |  |  | |  | |
| **4.** | **Ich erkenne den potenziellen Wert [der Intervention] für meine Arbeit.** |  |  |  |  |  | |  | |
| **5.** | **Es gibt Schlüsselpersonen, welche die Anwendung [der Intervention] vorantreiben und andere Personen einbeziehen.** |  |  |  |  |  | |  | |
| **6.** | **Ich finde, dass meine Beteiligung an [der Intervention] ein legitimer Teil meiner Rolle ist.** |  |  |  |  |  | |  | |
| **7.** | **Ich bin offen für neue Arten der Zusammenarbeit bei der Anwendung [(von) der Intervention]** |  |  |  |  |  | |  | |
|  |  | **1**  **Stimme überhaupt**  **nicht zu** | **2**  **Stimme nicht zu** | **3**  **Weder noch** | **4**  **Stimme zu** | **5**  **Stimme voll und ganz zu** | | **Nicht zutreffend** | |
| **8.** | **Ich werde [die Intervention] weiterhin unterstützen.** |  |  |  |  |  | |  | |
| **9.** | **Ich kann [die Intervention] leicht in meine bestehende Arbeit integrieren.** |  |  |  |  |  | |  | |
| **10.** | **[Die Intervention] wirkt sich negativ auf Arbeitsbeziehungen aus.** |  |  |  |  |  | |  | |
| **11.** | **Ich habe Vertrauen in die Fähigkeit anderer, [die Intervention] anzuwenden.** |  |  |  |  |  | |  | |
| **12.** | **Die Tätigkeiten werden Personen zugewiesen, welche über geeigneten Kompetenzen verfügen, [die Intervention] anzuwenden.** |  |  |  |  |  | |  | |
| **13.** | **Es werden ausreichend Schulungen angeboten, damit Mitarbeitende [die Intervention] anwenden können.** |  |  |  |  |  | |  | |
| **14.** | **Es sind ausreichend Ressourcen zur Anwendung [der Intervention] verfügbar.** |  |  |  |  |  | |  | |
| **15.** | **Die Führungskräfte unterstützen [die Intervention] angemessen.** |  |  |  |  |  | |  | |
| **16.** | **Mir sind Berichte über die Auswirkung [der Intervention] bekannt.** |  |  |  |  |  | |  | |
| **17.** | **Die Mitarbeitenden sind sich einig, dass [die Intervention] lohnenswert ist.** |  |  |  |  |  | |  | |
| **18.** | **Ich schätze die Auswirkungen [der Intervention] auf meine Tätigkeit.** |  |  |  |  |  | |  | |
|  |  | **1**  **Stimme überhaupt**  **nicht zu** | **2**  **Stimme nicht zu** | **3**  **Weder noch** | **4**  **Stimme zu** | **5**  **Stimme voll und ganz zu** | | **Nicht zutreffend** | |
| **19.** | **Rückmeldungen zu [der Intervention] können für zukünftige Verbesserungen genutzt werden.** |  |  |  |  |  | |  | |
| **20.** | **Ich kann anpassen, wie ich mit [der Intervention] arbeite.** |  |  |  |  |  | |  | |

**Vielen Dank, dass Sie diesen Fragebogen ausgefüllt haben.**

**[FRAGEBOGEN ENDE]**
