# Supplementary material for: Validation of the German Normalisation Process Theory Measure G-NoMAD: translation, adaptation, and pilot testing
Source: Implement Sci Commun. 2023 Oct 16;4:126. doi: 10.1186/s43058-023-00505-4 (PMC10578017; doi:10.1186/s43058-023-00505-4)
Supplement: Supplementary file 3 — Additional file 3. G-NoMAD manual. [file 43058_2023_505_MOESM3_ESM.docx]

| 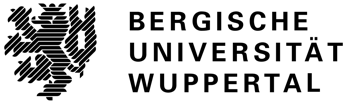 | **Bitte wie folgt zitieren:** Freund, J., Piotrowski, A., Bührmann, L., Oehler, C., Titzler., I., Netter, A., Potthoff, S., Ebert, D. D., Finch, T., Köberlein-Neu, J., Etzelmüller, A. (2023). Validation of the German Normalization Process Theory Measure G-NoMAD: Translation, Adaptation, and Pilot Testing. | 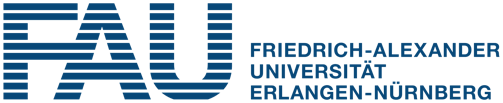 |
| --- | --- | --- |
| 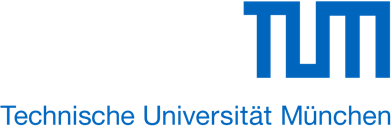 |  | 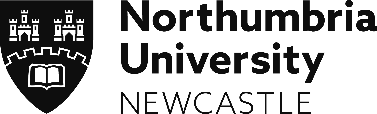 |

**Manual zur Nutzung des G-NoMAD**

[Dieser Abschnitt stellt eine Handlungsanleitung für Forschende dar, die Daten über den G-NoMAD Fragebogen erheben möchten. Dieser Teil wird nicht an die Studienteilnehmenden kommuniziert.]

**Vor der Nutzung des G-NoMAD Fragebogens empfehlen wir, die Instruktionen und einzelne Items des Fragebogens an ihren spezifischen Forschungs- und Anwendungskontext anzupassen.**

| 1. **Die Intervention** |
| --- |

Bitte beachten Sie, dass eine genaue Bezeichnung der Intervention in die dafür vorgesehenen Felder in eckigen Klammern einzutragen ist. Hierbei kann

es sich um eine bereits implementierte Intervention oder Arbeitsweise handeln.

Der Name sollte eine kurze und möglichst prägnante und genaue Beschreibung der Intervention oder Arbeitsweise darstellen und den Studienteilnehmenden bekannt sein. Die Intervention kann verschiedene Bereiche betreffen (z.B. eine neue Technologie, eine klinische Richtlinie, ein Medikament, ein Behandlungsverfahren etc.). Bei Bedarf können Sie den Namen der Intervention oder Arbeitsweise durch eine Beschreibung einführen und diese im Instruktionsteil des Fragebogens aufnehmen. Es kann hilfreich sein in der Beschreibung ebenso auf neue Prozesse Bezug zu nehmen, welche Sie als Bestandteile der Intervention (z.B. Einsteuerungsprozesse, Screening, Umsetzung einer neuen Technologie, Behandlungsverfahrens, etc.) erachten.

| 1. **Der Kontext** |
| --- |

Für eine korrekte Anwendung des G-NoMAD Fragebogens empfehlen wir Ihnen, dass Sie sich vorab ausreichend Gedanken über den spezifischen Kontext machen, in dem Sie die Implementierung einer neuen Intervention oder Arbeitsweise untersuchen wollen. Machen Sie sich ein genaues Bild von dem Kontext, in dem Ihre Zielgruppe den Fragebogen beantwortet und in welchem die Intervention oder Arbeitsweise implementiert wird. Es kann hilfreich sein, den spezifischen Implementierungskontext (z.B. Arztpraxis, Arbeitsgruppen, ambulantes oder stationäres Setting, o.Ä.) in der Instruktion konkret zu beschreiben.

| 1. **Die Personengruppen und Rollen** |
| --- |

Es kann vorkommen, dass Personen verschiedener Tätigkeitsbereiche an der Befragung teilnehmen, welche die Intervention oder Arbeitsweise auf unterschiedliche Weise nutzen oder auf verschiedene Arten von der Implementierung einer Intervention oder Arbeitsweise betroffen sind. In diesem Fall empfehlen wir, diesen unterschiedlichen Anwendungsbedingungen gerecht zu werden, indem die jeweiligen Rollenbeschreibungen den Fragebogeninstruktionen hinzugefügt werden. Dadurch soll es den Personen aus verschiedenen Rollen und Tätigkeitsfeldern erleichtert werden, die Fragebogen-Items im Kontext ihres Rollenbildes zu verstehen und beantworten zu können.

Eine mögliche Vorgehensweise könnte sein, die Zugehörigkeit zu einer der Gruppen zu Beginn des Fragebogens in einer Sektion zu sozio-demographischen Variablen abzufragen.

| 1. **Die Arbeitsweise oder Tätigkeit** |
| --- |

Der Fragebogen nutzt die Formulierung “Tätigkeiten” und “Arbeitsweisen”. In spezifischen Anwendungsfällen des NoMAD kann es sinnvoll sein, diese Wörter in Abhängigkeit des Kontextes im Fragebogen zu spezifizieren. Darüber hinaus ist es für die Interpretation des G-NoMADs je nach Forschungsfrage möglicherweise hilfreich, den Erfahrungsstand der Befragten mit der Intervention oder Arbeitsweise (z.B. Einschätzungen über die Häufigkeit oder die Dauer der Interventionsanwendung, o.Ä.) zu dokumentieren.

Hinweis: Da der Fragebogen für verschiedene Forschungskontexte individuell angepasst wird, empfehlen wir die genaue Formulierung der Items des in dem Forschungsvorhaben genutzten Fragebogens in der Publikation und/oder dem Studienbericht im Anhang/Supplement zu veröffentlichen und die Anpassungen und ihre Entscheidungsgrundlage in der Methode zu beschreiben.

| 1. **Hinweise zum Umgang mit der “nicht zutreffend” Antwortoption** |
| --- |

Die  Antwortoption “nicht zutreffend” bietet Aufschluss darüber, ob eine Frage nicht beantwortet werden konnte und verhindert eine Konfundierung

mit der mittleren Auswahl-Option, welche häufig von Studienteilnehmenden als neutrale Antwortoption aufgefasst wird. Um dennoch ausreichend Daten für die Analyse des Fragebogens zu erhalten, wird - ähnlich wie beim Umgang mit fehlenden Werten - ein Ersetzen des Wertes empfohlen. Dabei kann laut Huisman et al. (2000) der Personen-Mittelwert, d.h. der Mittelwert der beantworteten Items dieser Person innerhalb der jeweiligen Skala, verwendet werden. Wurden 50% oder mehr Items innerhalb einer Skala mit “nicht zutreffend” beantwortet, kann alternativ der Mittelwert des Items, d.h. der Mittelwert eines Items über alle Personen hinweg, verwendet werden.

**Literatur**

Huisman, M. (2000). Imputation of missing item responses: Some simple techniques. *Quality and Quantity*, 34, 331-351.
